# *In vitro* transdifferentiation of human peripheral blood mononuclear cells to photoreceptor-like cells

**DOI:** 10.1242/bio.016477

**Published:** 2016-05-11

**Authors:** Yukari Komuta, Toshiyuki Ishii, Makoto Kaneda, Yasuji Ueda, Kiyoko Miyamoto, Masashi Toyoda, Akihiro Umezawa, Yuko Seko

**Affiliations:** 1 Visual Functions Section, Department of Rehabilitation for Sensory Functions, Research Institute, National Rehabilitation Center for Persons with Disabilities, Tokorozawa, Saitama 359-8555, Japan; 2 Department of Physiology, Nippon Medical School, Sendagi, Bunkyo, Tokyo 113-8602, Japan; 3 ID Pharma Co. Ltd, Tsukuba, Ibaraki 300-2611, Japan; 4 Department of Vascular Medicine, Tokyo Metropolitan Institute of Gerontology, Itabashi-ku, Tokyo 173-0015, Japan; 5 Department of Reproductive Biology, Center for Regenerative Medicine, National Institute for Child Health and Development, Okura, Setagaya, Tokyo 157-8535, Japan

**Keywords:** Direct reprogramming, Human peripheral blood mononuclear cell (PBMC), Photoreceptor, Retinitis pigmentosa

## Abstract

Direct reprogramming is a promising, simple and low-cost approach to generate target cells from somatic cells without using induced pluripotent stem cells. Recently, peripheral blood mononuclear cells (PBMCs) have attracted considerable attention as a somatic cell source for reprogramming. As a cell source, PBMCs have an advantage over dermal fibroblasts with respect to the ease of collecting tissues. Based on our studies involving generation of photosensitive photoreceptor cells from human iris cells and human dermal fibroblasts by transduction of photoreceptor-related transcription factors via retrovirus vectors, we transduced these transcription factors into PBMCs via Sendai virus vectors. We found that retinal disease-related genes were efficiently detected in *CRX*-transduced cells, most of which are crucial to photoreceptor functions. In functional studies, a light-induced inward current was detected in some *CRX*-transduced cells. Moreover, by modification of the culture conditions including additional transduction of *RAX1* and *NEUROD1*, we found a greater variety of retinal disease-related genes than that observed in *CRX*-transduced PBMCs. These data suggest that *CRX* acts as a master control gene for reprogramming PBMCs into photoreceptor-like cells and that our induced photoreceptor-like cells might contribute to individualized drug screening and disease modeling of inherited retinal degeneration.

## Abstract (precis)

**Summary:** We established a method to generate photoreceptor-like cells from peripheral blood mononuclear cells by direct reprogramming to serve in disease modeling of inherited retinal degeneration.

## INTRODUCTION

Inherited retinal degenerative diseases deteriorate the quality of life (QOL) of patients mostly owing to a lack of efficient therapies. Owing to recent establishment of neural retina-induction in a self-organizing manner (Nakano et al., 2012; Ohlemacher et al., 2015), the disease-specific induced pluripotent stem cell (iPSC) model is thought to be ideal to overcome such incurable retinal diseases (Li et al., 2014; Schwarz et al., 2015). Induced photoreceptor cells generated from disease-specific iPSCs of retinitis pigmentosa (RP) patients were reported to reproduce pathogenic phenotypes (Jin et al., 2011, 2012; Phillips et al., 2014; Tucker et al., 2011; Yoshida et al., 2014). Although methods to generate photoreceptors from iPSCs have been established (Ohlemacher et al., 2015; Osakada et al., 2009), they are expensive and time-consuming.

For the past few decades, studies on transdifferentiation into other cell types have been performed (Freytag et al., 1994; Lassar et al., 1986; Weintraub et al., 1989; Xie et al., 2004); these have revealed a few transcriptional factors called master control genes (Lewis, 1992) that developmentally induce and maintain cell-specific gene expressions, lineages, and states (Takeuchi and Bruneau, 2009; Zhou et al., 2008). In most of these studies, source and target cells were derived from the same germ layer for developmental concepts. Direct reprogramming methods originated from these studies; therefore direct reprogramming methods are based on the database of cell-fate determining transcription factors, which can also directly reprogram somatic cells into other targeted cell types without the need for pluripotency (Feng et al., 2008; Ieda et al., 2010; Sekiya and Suzuki, 2011). Recently, other successful studies on transdifferentiation into other germ cell types were reported (Huang et al., 2011; Marro et al., 2011; Vierbuchen et al., 2010; Xie et al., 2004). Because direct reprograming is a fast and simple low-cost method of obtaining target cells, this method is potentially applicable to research on many human diseases.

We therefore employed the strategy of direct reprogramming to generate retinal photoreceptor cells from human somatic cells, defining a combination of transcription factors *CRX* (Morrow et al., 1999), *RAX* (Mathers et al., 1997) and *NEUROD* (Furukawa et al., 1997), that induce light responsive photoreceptor cells (Seko et al., 2012). In that study we induced ‘iris cells’ into photoreceptor cells. The iris and the retina share a common developmental origin. We then demonstrated that the same combination of genes used for human iris cells, i.e. *CRX*, *RAX* and *NEUROD*, generate human photoreceptor cells from human dermal fibroblasts and that additional *OTX2* (Nishida et al., 2003) gene transduction further amplifies the expression of retina-specific genes (Seko et al., 2014).

Though dermal fibroblasts are often utilized for direct reprogramming, sampling of adult human dermal biopsies actually requires surgical intervention and expertise. Recently, peripheral blood mononuclear cells (PBMCs) were used as a source of iPSCs (Kunisato et al., 2011; Seki et al., 2010; Staerk et al., 2010), and retinal cells were generated from human blood-derived iPSCs (Phillips et al., 2012). Indeed PBMC proliferation can be induced by IL-2, and these cells are easier and safer to harvest than dermal fibroblasts because collection of PBMCs does not require surgical intervention and expertise. Moreover, unlike that observed with fibroblasts, irrelevance of the origin difference of the donor's body to collect and the non-requirement of sampling expertise will reduce individual variations in PBMCs biopsies (Chang et al., 2002).

Here, based on the results of our studies using a direct reprogramming method to generate photoreceptors from human iris cells and dermal fibroblasts (Seko et al., 2014, 2012), we examined whether human PBMCs can be directly reprogrammed into photoreceptor-like cells *in vitro*.

## RESULTS

### PBMCs started to express cone-related genes sufficiently after transduction of *CRX* alone via Sendai virus vectors

**Fig. 1.**
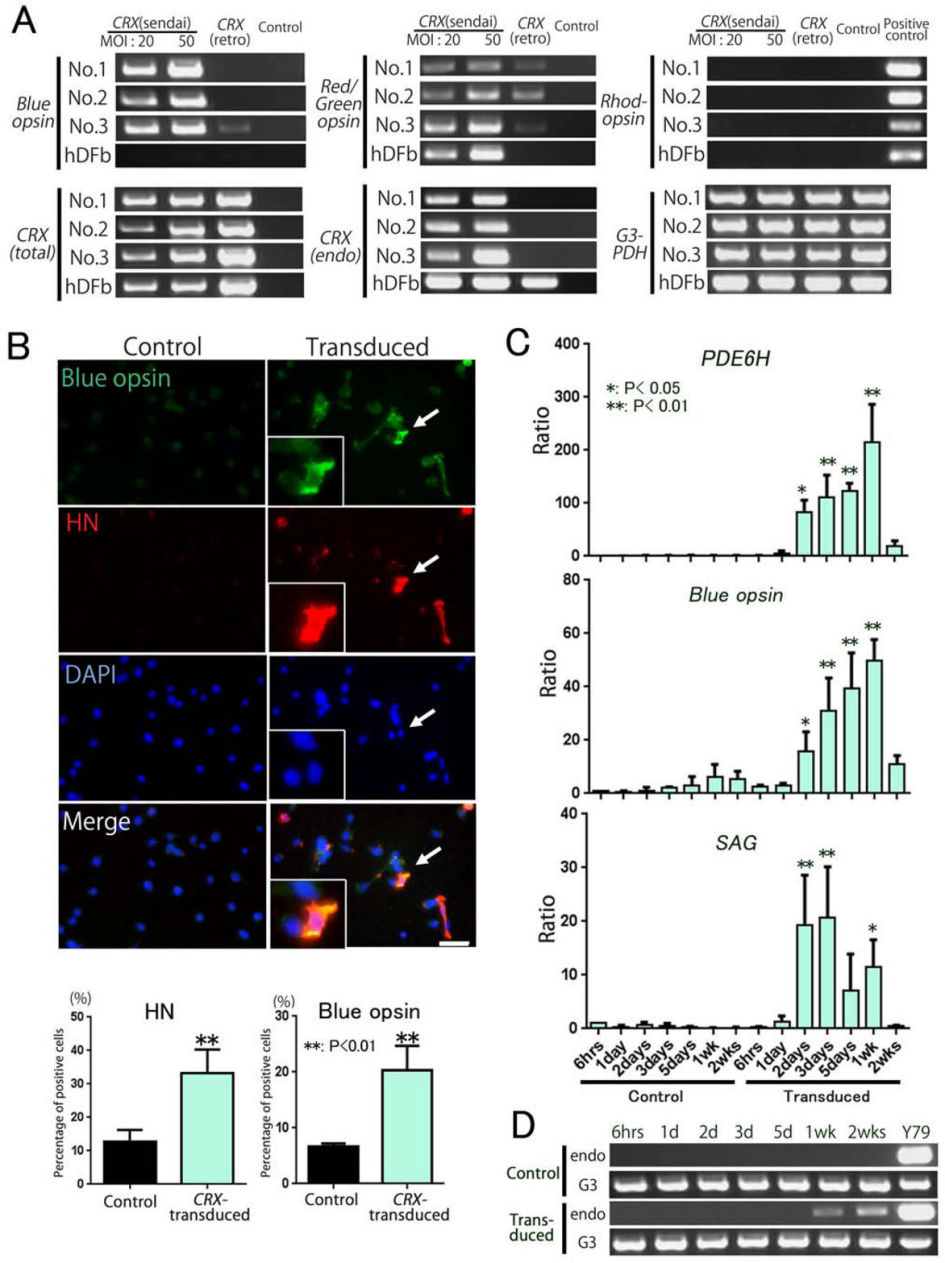
**PBMCs transduced with *CRX* via Sendai virus (SeV) expressed photoreceptor-related genes.** (A) Comparison between induced photoreceptor-related genes in *CRX*-transduced PBMCs and those in commercially available human dermal fibroblasts prepared by using SeV or retrovirus vectors. All PBMCs collected from the three volunteers were analyzed, and showed figures of blue opsin was detected from No. 2 PBMCs sample, red/green opsin was from No. 3 sample, rhodopsin was from No. 1 sample (nested PCR). Bands at the far right in the rhodopsin lanes show positive controls from the Y79 sample (positive control). *CRX* (total): total expression of *CRX* (35 PCR cycles); *CRX* (endo): endogenous expression of *CRX* (40 PCR cycles); *G3PDH*: housekeeping gene as internal control. (B) Immunocytochemistry and populations of cells expressing blue opsin (green) or HN (red). White arrows indicate a representative double-stained cell (enlarged in the left square). Nuclei were stained with DAPI (blue). Scale bar: 20 μm. Populations of blue opsin- or HN-positive cells were displayed in graphs (bottom). (*n*=4, **P<0.01; Student's *t*-test). (C) Temporal patterns of detected photoreceptor-related genes *PDE6H*, blue opsin, and *SAG* determined by real-time PCR. No. 2 PBMCs were analyzed for 6 h, 1 day, 2 days, 3 days, 5 days, 1 week and 2 weeks after *CRX* transduction. Samples from controls at 6 h were used as references (*n*=3; *P<0.05, **P<0.01; Dunnett's test). (D) Induced endogenous *CRX* expression. Specific primer sets were designed to distinguish endogenous from exogenous expression of CRX. Endogenous *CRX* expression was detected in *CRX*-transduced PBMCs at 1 week after transduction (endo: endogenous *CRX*, G3: *G3PDH*). All error bars represent s.d.

Expression of photoreceptor-related genes was examined by RT-PCR 7 days after transduction of the *CRX* gene alone by retrovirus vectors or Sendai virus vectors (SeV vectors) in PBMCs isolated from the blood of three healthy donors, designated No. 1-3. We found that the *CRX* gene was effective in inducing photoreceptor-related genes, blue opsin and red/green opsin, in the PBMCs. After transduction of the *CRX* gene by SeV-*CRX* at 20 or 50 MOI, PBMCs efficiently expressed the blue opsin and red/green opsin genes (Fig. 1A). When PBMCs were transduced with retrovirus vectors, the blue opsin gene was not detected. The red/green opsin gene was specifically expressed in cone-photoreceptor cells. Compared with that observed in PBMCs, human dermal fibroblasts expressed these photoreceptor-related genes at a much lower level following transduction of *CRX* alone via retrovirus or SeV vectors. However, rhodopsin was not detected following transduction of *CRX* alone.

Induction of the expression of photoreceptor-related proteins was studied by immunocytochemistry (Fig. 1B,Fig. S1). On the third day after transduction, blue opsin-positive cells in the No. 2 PBMCs were counted (Fig. 1B). Among the *CRX*-transduced cells, 20.4% were PBMCs bearing blue opsin signals and HN-positive cells (HN, hemagglutinin-neuraminidase glycoprotein used as Sendai virus infection marker) accounted for 90.9% of blue opsin-expressing cells and 33.2% of the total cells. Among the control cells, blue opsin-expressing cells accounted for 6.7% of the total cells and HN-positive cells accounted for 12.7% of the total cells. HN- or blue opsin-expressing control cells were too few to reliably calculate the percentage of HN-positive cells in blue opsin-expressing cells. These results demonstrated that *CRX*-transduced cells efficiently expressed blue opsin. However, *CRX*-transduced cells exhibited round shape, unlike genuine photoreceptors. To investigate the time course of photoreceptor-related gene expression levels after *CRX*-transduction, *PDE6H* (phosphodiesterase 6H, CGMP-specific, cone, gamma), blue opsin, and *SAG* were evaluated by quantitative real-time PCR, using sequentially harvested *CRX*-transduced No. 2 PBMCs (Fig. 1C). All of the genes were upregulated 2 days after transduction. Expression of *PDE6H* and blue opsin genes peaked 1 week later. At 2 weeks after transduction, expression levels of all these genes declined to very low or undetectable levels. We also confirmed that the expression of endogenous *CRX* genes was detected at 1 week and 2 weeks after transduction (Fig. 1D). These results showed that at least some cells transduced with *CRX-*SeV were reprogrammed to form photoreceptor-like cells within approximately 1 week.

### Induced photoreceptor-like cells from PBMCs showed photoresponse *in vitro*

**Fig. 2.**
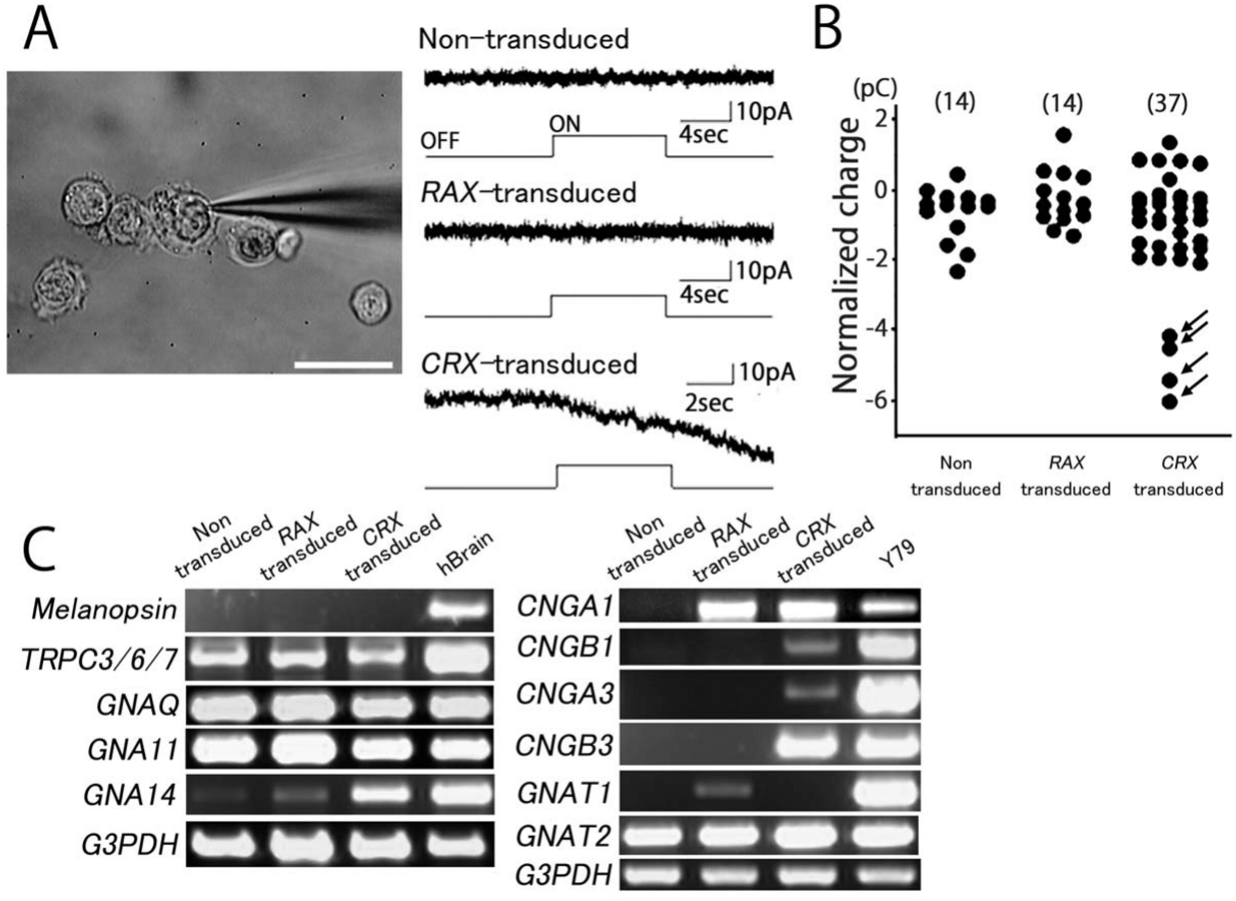
**Responses to light in control 1 (non-transduced cells), control 2 (*RAX1*-transduced cells), and *CRX*-transduced cells.** (A) Responses to light in control 1 (non-transduced cells, top), control 2 (*RAX1*-transduced cells, middle), and *CRX*-transduced cells (bottom). No. 2 PBMCs were used. Timing and duration of light stimulation are shown under the current trace. Holding potential was −40 mV. Scale bar: 20 μm. (B) Summary of the light responses. Amplitude of the responses was normalized to the total charge. Details of the data analysis are provided in Fig. S4. (C) Expressions of phototransduction-related genes in *RAX1*- or *CRX*-transduced PBMCs. Expression of melanopsin cascade genes (melanopsin, *TRPC*s, *GNAQ*, *GNA11* and *GNA14*) and cone/rod photoreceptor-related opsin cascade (phototransduction cascade) genes (*CNGA1*,*3*, *CNGB1*,*3* and *GNAT1*,*2*) was analyzed. Each positive control was extracted from the human brain (Ambion) or Y79 cell line. Downstream genes of the melanopsin cascade were abundantly expressed in controls and *RAX1*- and *CRX*-transduced PBMCs.

We examined whether the *CRX*-transduced No. 2 PBMCs responded to light stimuli. In some cells, we could record a detectable light-induced inward current, although light-induced responses were undetectable in most cells (Fig. 2A, bottom panel; Fig. 2B). In contrast, no detectable inward current was observed in control 1 (non-transduced cells) or control 2 (*RAX1*-transduced cells) in all cells examined (Fig. 2A). To confirm whether the inward current observed in *CRX*-transduced human PBMCs was really triggered by light stimuli, we calculated the total charge generated during light stimulation (Fig. 2B). In control 1 and control 2, light responses showed a maximum charge of ±3 pC. Since control 1 and control 2 did not express any markers for photoreceptors and intrinsically photosensitive retinal ganglion cells (ipRGC) at the mRNA level, light responses with a charge less than ±3 pC were considered to reflect a fluctuation of the baseline during the recordings. Distribution of light responses of *CRX*-transduced PBMCs was classified into distinct two clusters. Light responses in most *CRX*-transduced cells (33 out of 37) were less than ±3 pC, suggesting that they were not photosensitive (Fig. 2B). In four cells of *CRX*-transduced cells, we observed light responses higher than −3 pC (Fig. 2B, arrow). Therefore, we concluded that reprogramming at the functional level probably occurs only in some parts of the *CRX*-transduced human PBMCs. We further examined whether the light responses in *CRX*-transduced human T-cell were reproducible in other donors (No. 1 and No. 3). In both donors, *CRX*-transduced PBMCs responded to light stimuli, while no detectable inward current was observed in non-transduced PBMCs (Fig. S3). As mentioned in No. 2 PBMCs, efficiency of reprogramming is not so high in both donors' PBMCs (3 out of 15 cells for No. 1, 2 out of 14 cells for No. 3; arrow).

These results were confirmed by RT-PCR of phototransduction-related genes (Fig. 2C). Photostimulation of the rod or cone pathway produces hyperpolarizing responses, while activation of the melanopsin pathway produces depolarizing responses (Hattar et al., 2002). In the present study, melanopsin was not detected in controls or *CRX*-transduced cells. Downstream genes of the melanopsin cascade, TRPCs (transient receptor potential cation channel, subfamily C) and Gqα subunits [*GNAQ* (guanine nucleotide binding protein (G protein), *GNA11* (G protein, Alpha 11), and *GNA14* (G protein, Alpha 14)] (Hughes et al., 2015) were abundantly expressed in both controls and *CRX*-transduced cells; in particular, *GNA14* was detected in *CRX*-transduced cells. By contrast, expression of phototransduction-related genes, CNGs, and Gαt subunits (*GNAT1* and *GNAT2*) was relatively insufficient. Though *GNAT2* [guanine nucleotide-binding protein G(T), alpha-2 subunit] was sufficiently expressed in all samples, *CNGA1* (cyclic nucleotide gated channel alpha 1) was slightly expressed in controls and *CRX*-transduced cells. *CNGB1* and *GNAT1* [guanine nucleotide-binding protein G(T), alpha-1 subunit] were not detected, while *CNGA3* and *CNGB3* were detected in *CRX*-transduced cells; however *CNGA3* expression was very low.

### Numerous retinal disease-related genes were expressed in photoreceptor-directed PBMCs transduced with *CRX*

**Fig. 3.**
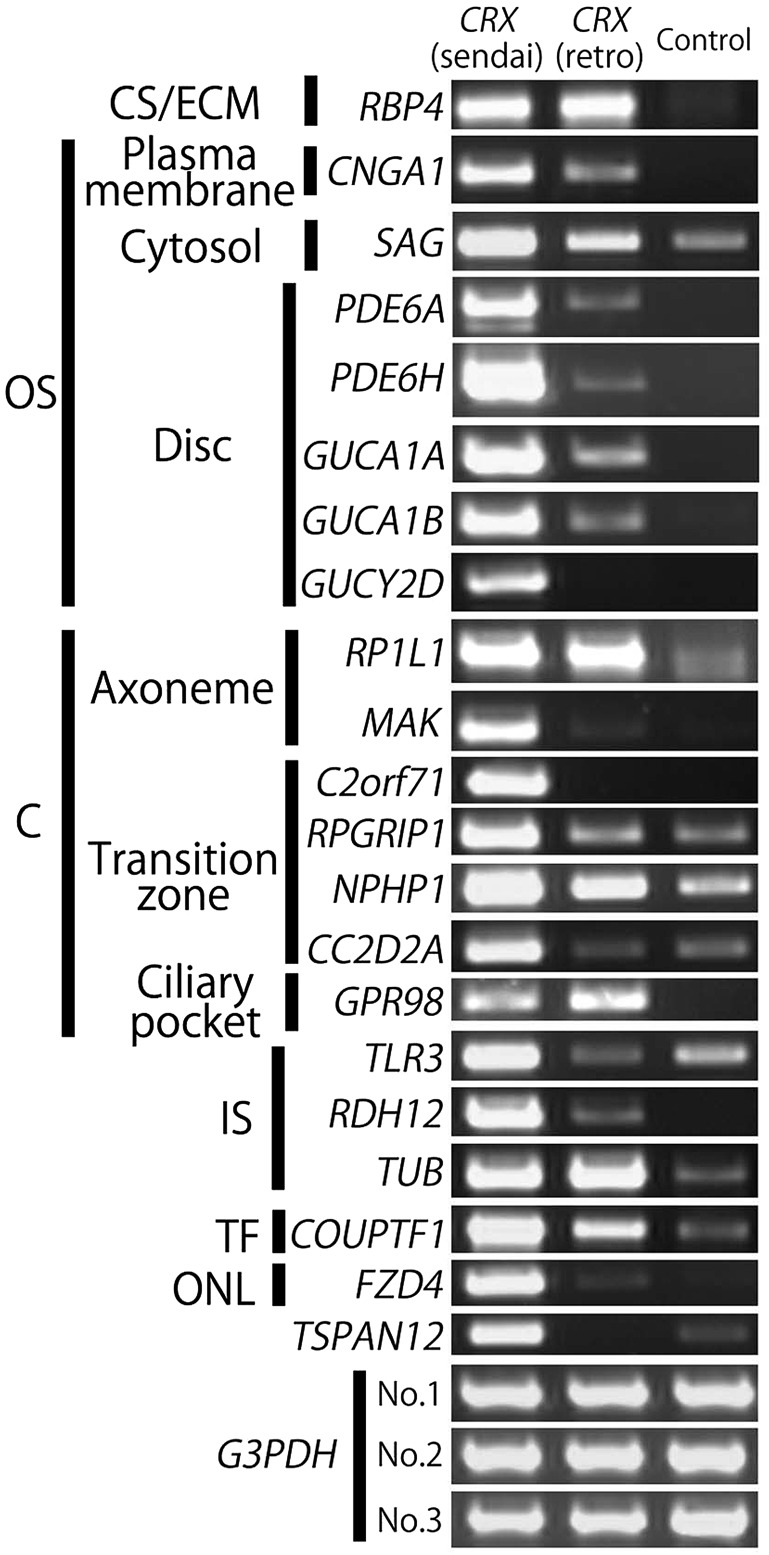
**Many retinal disease-related genes were induced in *CRX*-transduced PBMCs prepared by SeV.** Expression of retinal disease-related genes in *CRX*-transduced and control (non-transduced) PBMCs by RT-PCR. Results of *PDE6H*, *CNGA1*, *RPGRIP1*, *C2orf71*, *CC2D2A*, *COPTF1* and *TSPAN12* are from No. 1 PBMCs sample, those of *RBP4*, *GUCA1A*, *GUCA1B*, *GUCY2D*, *PDE6A*, *SAG* and *GPR98* are from No. 2 sample, and those of *RP1L1*, *MAK*, *NPHP1*, *TLR3*, *RDH12*, *TUB* and *FZD4* are from No. 3 sample. PBMCs transduced with *CRX* via SeV vectors more efficiently induced retinal disease-related genes than those transduced with *CRX* via retrovirus vectors, and most of the detected genes were related to photoreceptor functions. CS/ECM, cytoplasm/extracellular matrix; OS, outer segment; C, cilia-related; IS, inner segment; ONL, outer nuclear layer; TF, transcription factor.

**Fig. 4.**
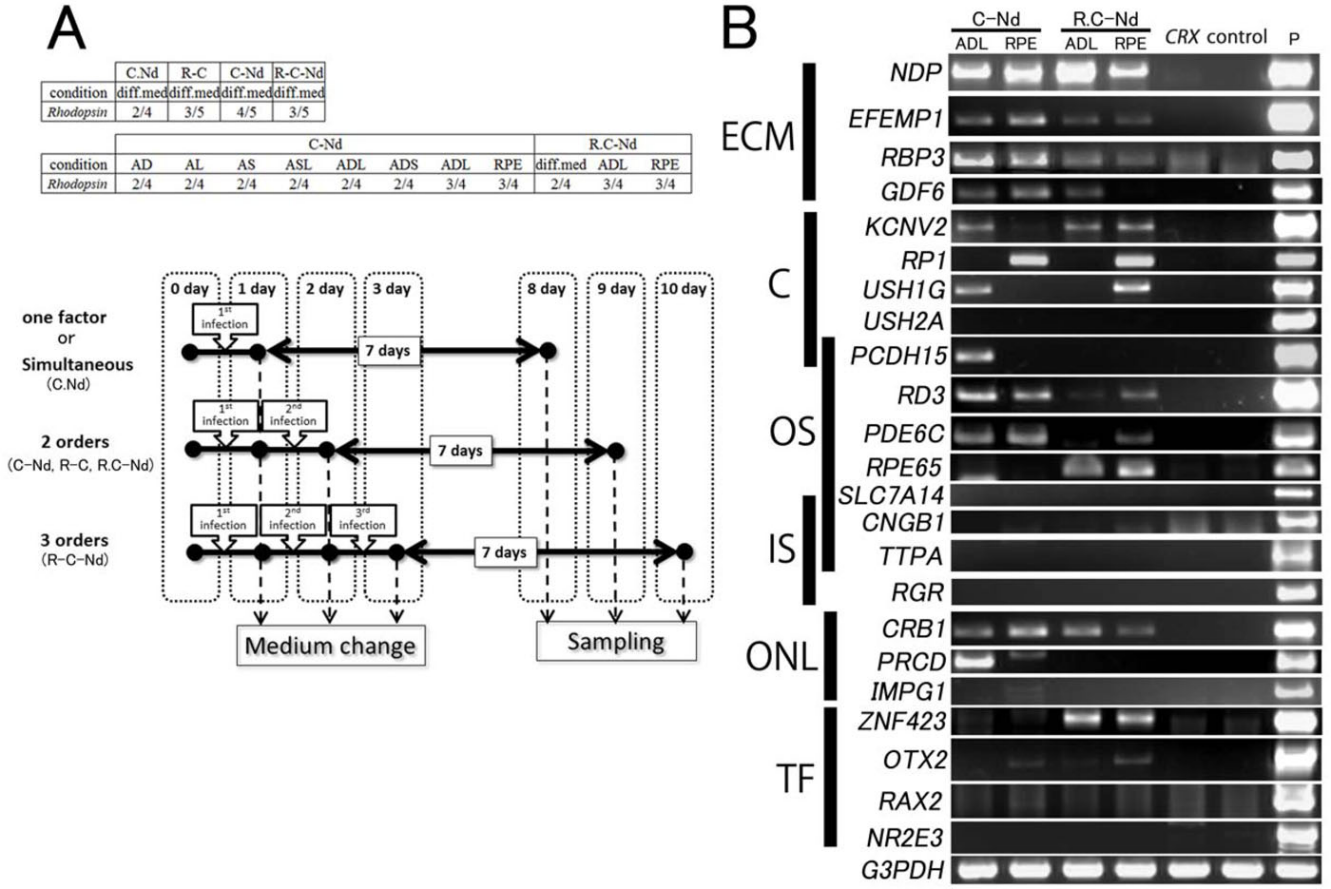
**Transduction of *RAX1* and *NEUROD1* in addition to *CRX*, with incubation in a modified medium improved the expression of retinal disease-related genes.** (A) Relatively effective modified conditions and frequencies of rhodopsin detection. Modified conditions tended to induce rhodopsin expression. Nos 1-3 PBMCs were used for the upper table, while No. 2 PBMCs were used for the lower table. The numerators show the number of experiments in which rhodopsin expression was detected, and the denominators show the total number of inductions. C, *CRX*; R, *RAX1*; Nd, *NEUROD1*. The schematic shows the experimental protocol, with transduction and culture conditions indicated by symbols; for example, C.Nd, *CRX* and *NEUROD1* were simultaneously transduced, R.C-Nd, *RAX1* and *CRX* were simultaneously transduced followed by transduction of *NEUROD1*. A, Activin A added; D, DKK1 added; L, Lefty2 added; RPE, including RPE-conditioned medium; diff.med, unmodified differentiation medium. (B) Detection of some retinal disease-related genes expression that was absent in PBMCs transduced with *CRX* alone. Positive controls were derived from the human brain (Ambion) or Y79 cell line (human brain samples were used for *NDP*, *PCDH15* and *TTPA*). ECM, extracellular matrix; OS, outer segment; C, cilia-related; IS, inner segment; ONL, outer nuclear layer; TF, transcription factor.

Because retinal disease-related genes (listed on the RetNet website; https://sph.uth.edu/retnet/, defects of these genes are associated with retinal diseases) are considered to manage important functions of photoreceptor cells, we analyzed the expression of some retinal diseases-related genes in *CRX*-transduced Nos 1-3 PBMCs by RT-PCR to investigate the applicability of our induced photoreceptor-like cells for basic and clinical studies on photoreceptor cells. Detected genes are indicated in Fig. 3. The expression of most of these genes was more efficiently increased in *CRX*-transduced cells prepared using SeV vectors than in those prepared using retrovirus vectors. Most retinal disease-related genes that were detected in *CRX*-transduced cells are specifically expressed in photoreceptor cells (Cremers et al., 2002; Ikeda et al., 2002; Rachel et al., 2012; Roosing et al., 2014; Smith et al., 2009; Trifunović et al., 2008; Yu and Hazlett, 2006). Among genes that were detected by *CRX* transduction, retinal disease-related genes such as *GUCA1A* (guanylate cyclase activator 1A), *GUCA1B* (guanylate cyclase activator 1B), *GUCY2D* (guanylate cyclase 2D), *PDE6A* (phosphodiesterase 6A, CGMP-specific, rod, alpha), *PDE6H*, *SAG*, *CNGA1*, *CNGA3* and *CNGB3* are ordinarily expressed in the retinal outer segments, *RP1*, *RP1L1*, *MAK* (male germ cell-associated kinase), *RPGRIP1* (retinitis pigmentosa GTPase regulator interacting protein 1), *NPHP1* (nephronophthisis 1), *C2orf71* (chromosome 2 open reading frame 71), *CC2D2A* (coiled-coil and C2 domain containing 2A), and *GPR98* (ADGRV1; adhesion G protein-coupled receptor V1) are expressed in the cilia, *TLR3*, *RDH12* and *TUB* (tubby bipartite transcription factor) are expressed in the retinal inner segments, and *FZD4* is expressed in the inner nuclear layer. Expression of the transcription factor *COUPTF1* increased as well. *TSPAN12* (Tetraspanin 12) was also detected but was previously reported to be expressed in the retinal vascular endothelial cells (Tummala et al., 2010). Genes that remained undetected here were analyzed again in Fig. 4 under improved culture conditions.

### Several retinal disease-related genes that were not detected in *CRX*-transduced PBMCs after transduction in the order of *RAX1* and *CRX* followed by *NEUROD1* were expressed

Expression of the rhodopsin gene was not detected in PBMCs transduced with *CRX* alone. Because effective photoreceptor-directed induction by transduction of a combination of *CRX*, *RAX1*, and *NEUROD1* (Seko et al., 2014, 2012) has been reported using human iris cells and human dermal fibroblasts, we transduced these genes by using SeV vectors in this experiment. We examined whether transduction of additional transcription factors, *RAX1* and *NEUROD1,* or modifications of the differentiation medium could increase the expression levels of retinal disease-related genes. We analyzed rhodopsin expression by nested PCR, using induced photoreceptor-like cells at 1 week after the first transduction by using Nos 1-3 PBMCs (Fig. 4A). In Fig. 4A, relatively effective conditions and the frequencies of rhodopsin detection are listed. *RAX1* and *NEUROD1* did not trigger the expression of rhodopsin or blue opsin. *NEUROD1* repressed the inducible effect of *CRX*, particularly when transduced before *CRX*; hence, *NEUROD1* was transduced simultaneously with *CRX* or after *CRX*. Photoreceptor differentiation was the most effective when *CRX* was transduced first, followed by transduction of *NEUROD1* 1 or 2 days later, or when *RAX1* and *CRX* were transduced simultaneously first, followed by transduction of *NEUROD1* 1 or 2 days later.

We also examined the effects of modification of the differentiation medium including all combinations of Activin A (100 ng/ml), Dkk (100 ng/ml), Shh (200 ng/ml), Lefty2 (500 ng/ml), and the conditioned medium of primary cultured retinal pigment epithelium (RPE) cells of rats by half. In these modified media, *CRX* was transduced into No. 2 PBMCs first, followed by *NeuroD1* 1 or 2 days later and expression of rhodopsin was analyzed 1 week after *CRX* transduction (Fig. 4A). Gene expression of rhodopsin was more frequently detectable in modified medium containing Activin A, Dkk, and Lefty2 or in the RPE-conditioned medium. These results suggest that such modifications facilitate the differentiation of human PBMCs into photoreceptor-like cells.

**Fig. 5.**
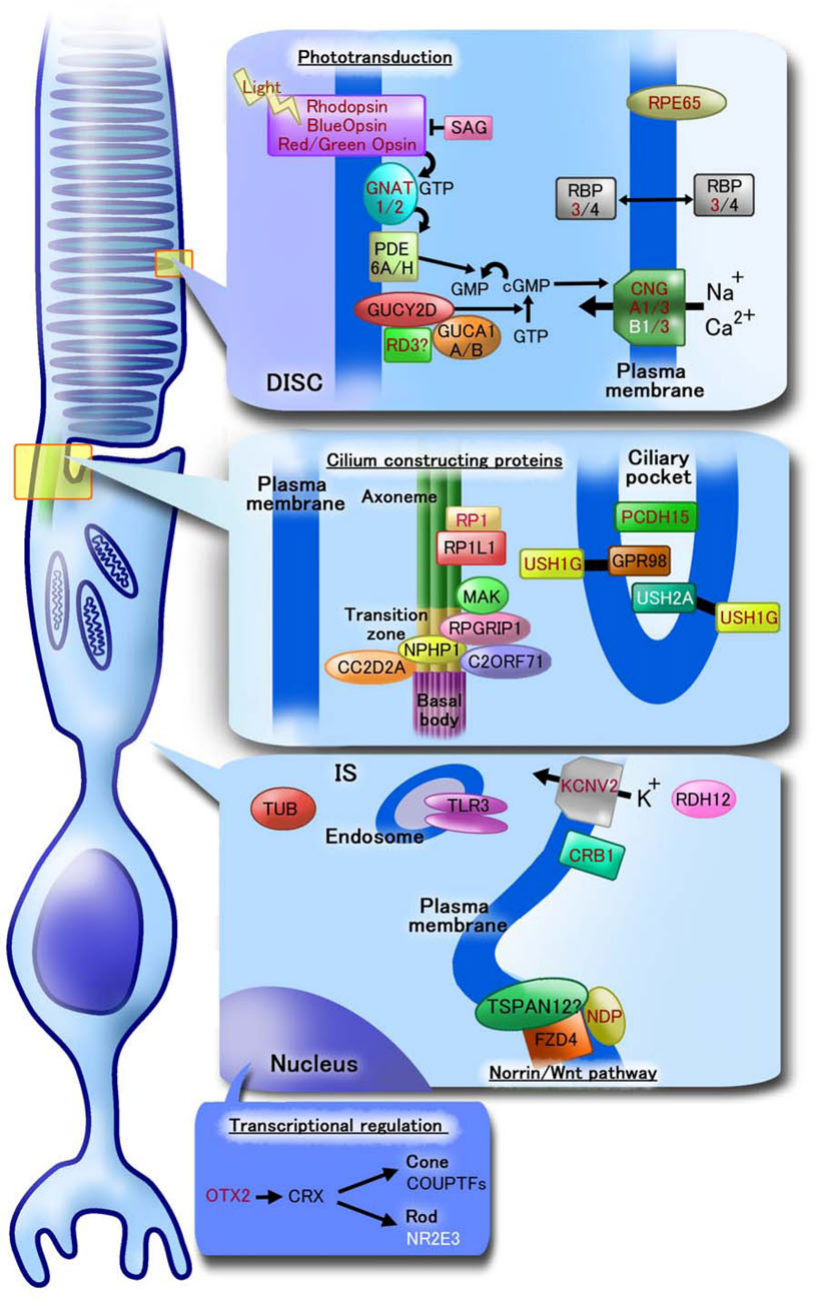
**An illustration** **showing roles or functions of analyzed retinal disease-related genes.** Localization and functions of analyzed retinal disease-related genes are illustrated based on previously reported papers. Gene names written in black and red here indicate that expression of those genes was detected in *CRX*-transduced PBMCs and in modified culture conditions, respectively (Figs. 3,4). Expression of genes written in white was not detected in any culture conditions. Localization is categorized into disc, cilium, inner segment (IS) and nucleus. Phototransduction: in opsins (rhodopsin, blue opsin, red/green opsins), 11-cis retinal is isomerized to all-trans-retinal by absorption of photons. All-trans-retinol is converted from all-trans-retinal by RDH12 and carried by RBP3/4. Activated opsins in turn activate G protein transducin α subunit (GNAT1/2), and GNAT1/2 activates PDE6A/H. PDE6A/H hydrolyses cGMP to GMP. cGMP is synthesized by guanylyl cyclase (GUCY2D), which reaction is regulated by guanylyl cyclase activating protein (GUCA1A/B) and RD3. CNG channels are composed of CNGA (CNGA1/3) and CNGB (CNGB1/3) subunits. CNG channels are kept open by cGMP for influx of potassium and calcium ion (den Hollander et al., 2010; Larhammar et al., 2009). RBP3 (equal to IRBP) and RBP4 bind and protect 11-cis or all-trans retinoids, and transport these retinoids between the retinal pigment epithelium and photoreceptors (den Hollander et al., 2009). RPE65 is expressed in mammalian cone cells as well as retinal pigment epithelium and synthesize 11-cis-retinol from all-trans-retinyl ester (Znoiko et al., 2002). RGR (retinal G protein coupled 2eceptor) binds all-trans-retinal and converts it to 11-cis retinal (Wenzel et al., 2005). RDH12 (retinol dehydrogenase 12) was reported to play roles for retinoid cycle (Maeda et al., 2006). Cilium constructing proteins: RP1, RP1L1, MAK, RPGRIP1, NPHP1, CC2D2A and C2ORF71 are relating to ciliogenesis and/or function of the photoreceptor cilium. Those were reported about their expressions around transition zone (Rachel et al., 2012). Ectodomains of USH2A (Aliases are Usherin or USH2) and GPR98 (equal to ADGRV1, USH2B, USH2C, VLGR1) connect USH1G (equal to SANS) ant these complexes construct USH protein network and are related to vesicle delivery along microtubules. PCDH15 (equal to USH1F or CDHR15) is essential for maintenance photoreceptors (Maerker et al., 2008; Sahly et al., 2012). Others: KCNV2 is voltage-gated potassium channel subunit (Wu et al., 2006). CRB1 (Crumbs homolog 1) constructs zonula adherens and maintains photoreceptor morphogenesis (Pellikka et al., 2002). The autosomal recessive mutation tubby (tub), which show retinal degeneration, occurred spontaneously in C57BL/6J (B6) mice (Sahly et al., 1998). TLR3 (toll-like receptor 3) protect photoreceptors from oxidative stress (Patel and Hackam, 2014). Norrin, FZD4 and TSPAN12 have been suggested to participate in Norrin signal pathway and their protective roles from cell death by light damage (Braunger et al., 2013). OTX2 and CRX are expressed in photoreceptor precursors (Furukawa et al., 1997; Nishida et al., 2003). NR2E3 promotes differentiation to rod photoreceptors (Cheng et al., 2004) and COUPTFs regulate S- or M-opsin expression (Satoh et al., 2009).

Several retinal disease-related genes absent in cells transduced with *CRX* alone were also significantly detected (Fig. 4B, Fig. 5) in cells transduced with *CRX* before *NeuroD1* or those transduced with *RAX1*+*CRX* before *NeuroD1* in a modified medium containing Activin A, Dkk, and Lefty2 or the RPE-conditioned medium. The detected genes included *CNGB1*, *EFEMP1* (EGF containing fibulin-like extracellular matrix protein 1), *GDF6* (growth differentiation factor 6) (Asai-Coakwell et al., 2013), *KCNV2* (potassium channel, voltage gated modifier subfamily V, member 2), *NDP* (Norrie disease protein, Norrin) (Braunger et al., 2013), *OTX2*, *RBP3*, *RD3* (retinal degeneration 3) (Azadi, 2013), *RPE65* (Cremers et al., 2002; Znoiko et al., 2002), *USH1G* (Maerker et al., 2008; Sahly et al., 2012), and *ZNF423* (zinc finger protein 423). However, *NR2E3*, *RAX2*, *RGR*, *SLC7A14*, *TTPA* [tocopherol (alpha) transfer protein] and *USH2A* (Usher syndrome 2A) were not detected following the treatments carried out in this study.

## DISCUSSION

We found that retinal disease-related genes, most of which are crucial to photoreceptor functions, were efficiently expressed in PBMCs transduced with *CRX* via SeV vectors. In functional physiological studies, light-responsive cells could be found among *CRX*-transduced cells. Moreover, by modification of culture conditions, a greater variety of retinal disease-related genes was detected in photoreceptor-like cells generated from PBMCs.

PBMCs started to express cone-photoreceptor-related genes after transduction of *CRX* alone using SeV vectors. Blue opsin and red/green opsin were more efficiently and intensely expressed in *CRX*-transduced PBMCs prepared using SeV vectors than in those prepared using retrovirus vectors (Fig. 1A, Fig. 3) because transduction by SeV vectors could be more efficient than that by retrovirus. However the expression level of the blue opsin gene increased in *CRX*-transduced PBMCs but not in fibroblasts, although transduction was performed by SeV vectors in both cells. Endogenous *CRX* expression was detected in dermal fibroblasts transduced with *CRX* by both retrovirus and SeV vectors, but was detected in PBMCs transduced only by SeV vectors. These differences might be attributed to variable reprogramming efficiencies based on different methylation signatures dependent on cell types as previously reported (Bramswig et al., 2013; Nishino et al., 2011). We also confirmed that the expression of endogenous *CRX* genes was detected at 1 week and 2 weeks (Fig. 1A,D). This result shows that at least some *CRX-*SeV transduced cells were reprogrammed to photoreceptor-like cells within approximately 1 week. Time-course analysis revealed that upregulated expression of the blue opsin and PDE6H genes in *CRX*-transduced cells prepared by SeV vectors peaked at 1 week after transduction and declined by 2 weeks after transduction (Fig. 1C). Expression of S-antigen was detected and peaked immediately after transduction. The observed decline of gene expression might be attributed to the fact that the gene transduced by SeV vectors is not inherited in the daughter cells, causing a relative decrease in the population of the transduced cells. Therefore, if a longer incubation was needed, a sorting system should be established to separate transduced cells.

Induced photoreceptor-like cells from PBMCs were photoresponsive *in vitro*, at least in part. To investigate whether our induced photoreceptor-like cells from PBMCs have the functional nature as well as their genes expression profiling of photoreceptors, we examined whether *CRX*-transduced No. 2 PBMCs responded to light stimuli. In a few cells, we could record a detectable light-induced inward current although light-induced responses were not detectable in most cells (Fig. 2A, bottom; Fig. 2B). This scarcity of light-responsive cells raised a possibility that the observed light-induced inward current in these cells might have been generated by an occasional deflection due to cell damage such as that following viral transduction. However this is not likely the case owing to the following reasons: first, the occasional deflection in non-transduced cells was limited to ±3 pC from the baseline. Second, the occasional deflection in *RAX*-induced cells was also limited to ±3 pC from the baseline, indicating that viral transduction itself did not increase the amplitude of occasional deflection. In the present study, as we judged cells with normalized total charge higher than 3 pC as light-responsive, it is likely that functional reprogramming of *CRX*-transduced cells occurs in limited populations. Such a scarcity of light responsive cells was repeatedly observed in the other two cell lines. The low frequency of light responses in reprogrammed *CRX*-transduced cells might be explained by the possibility that only a part of blue opsin-expressing cells possess the mature signaling cascade to evoke light responses, the small size of the cells, efficiency of protein expression, etc.

In this study, we detected light-induced inward current and could not detect the typical outward current of photoreceptors. Since the light-induced inward current seemed to be mediated by melanopsin-associated phototransduction as observed in iris-derived photoreceptor-like cells (Hartwick et al., 2007; Hattar et al., 2002; Seko et al., 2012), we investigated the expression of melanopsin by RT-PCR. However, expression of melanopsin was not detected in photoreceptor-directed PBMCs. We therefore examined photoreceptor-related and melanopsin-related genes that function in phototransduction. We detected strong expression of downstream genes of the melanopsin cascade such as TRPC and Gqα, but not GNA14, which are commonly expressed in the control and *CRX*-transduced cells, indicating expression of these genes resulted from the original PBMCs. *CNGB3* expression was detected in abundance in *CRX*-transduced cells, but *CNGA3*, which collaborates with CNGB3, was not sufficiently expressed. This fact might be the reason why the phototransduction cascade could not mediate the light stimuli as the typical outward current of photoreceptors. Proteins involved in the signal transduction cascade of melanopsin, such as TRPC and Gqα proteins, which induce depolarization, were expressed abundantly while Gαt and CNG proteins, which induce hyperpolarization, were not sufficiently expressed. The reason why an inward current was detected in *CRX*-transduced cells expressing photoreceptor-related genes is unknown at this time; however it might be possible that signals passed from blue, red, or green opsin to a downstream point in the melanopsin signaling cascade in *CRX*-transduced cells, leading to the production of depolarization by light stimuli. In a future study we will investigate the presence of a novel ‘phototransduction’ cascade in *CRX*-transduced cells.

We found that numerous retinal disease-related genes were detected in *CRX*-transduced PBMCs, and most of them were reported to be expressed in photoreceptors, in which they play essential roles (den Hollander et al., 2010; Rachel et al., 2012; Smith et al., 2009; Trifunović et al., 2008). We did not evaluate the expression of several retinal disease-related genes such as *UNC119* and *BBS5* because of their strong expression in primary PBMCs (Fairfax et al., 2012; Zhao et al., 2014) or their ubiquitous expression in other regions besides the retina. Furthermore, we found a greater variety of retinal disease-related genes by modification of culture conditions compared to that observed in *CRX*-transduced PBMCs. Transduction of *RAX1* and *NeuroD1* in addition to *CRX* and modification of the differentiation medium facilitated induction of a greater variety of retinal disease-related genes. Improvement of induction of retinal disease-related genes expression by additional transduction of *RAX1* and *NEUROD1* supports our previous reports, in which those three factors are essential for generation of photoreceptor-like cells from human somatic cells (Seko et al., 2014, 2012). Although *RAX1* and *NEUROD1* are not master genes of photoreceptors because they failed to induce major photoreceptor markers such as rhodopsin and blue opsin by themselves, they would be essential for differentiation of cells into rod-photoreceptors. However, several photoreceptor-related genes were not detected and the expression level of the rhodopsin gene was very low in photoreceptor-directed PBMCs; hence other factors may be necessary for differentiation of cells into mature photoreceptor cells. The round morphology of the cells we generated, the deficiency of expression of several photoreceptor-related genes, and the difference in the direction of photo-response indicate that our cells are not completely differentiated. However, the fact that our immature photoreceptor-like cells express many retinal disease-related genes indicates our methods may be useful for the study of the target RNAs or proteins which are expressed in human retina, and analysis of abnormalities in signaling, mRNA sequence or protein structure (Fig. 5).

In conclusion, PBMCs, whose proliferation is induced by IL-2, are collected in a much easier manner and are safer to use compared to dermal fibroblasts; these cells have potential for use as a cell source of differentiation into photoreceptors. *CRX* transduced by SeV acts as a master control gene for reprogramming of PBMCs into photoreceptors and the induced photoreceptor-like cells we generated might contribute to individualized drug screening and disease modeling of inherited retinal degeneration.

## MATERIALS AND METHODS

### Isolation and culture of human PBMCs

PBMCs were extracted from 5 ml samples of blood from three healthy volunteers under the approval of the Ethics Committee of the National Rehabilitation Center for Persons with Disabilities (NRCPD). Signed informed consent was obtained from three healthy donors (48 year-female, 63 year-male, and 53 year-male), and samples were irreversibly de-identified. All experiments involving human cells and tissues were performed in line with the Declaration of Helsinki. PBMCs were isolated using BD Vacutainer^®^ Cell Preparation Tube according to the manufacture's recommended procedure (BD Pharmingen, San Diego, CA, USA) (Corkum et al., 2015), and the three samples were designated as No. 1, No. 2, and No. 3 PBMC, respectively. Collected PBMCs were suspended, cultured and expanded in an IL-2 containing culture medium (KBM502, Kohjin Bio, Saitama, Japan) on anti-CD3 antibody-coated (BD Pharmingen) 10 cm dishes. Proliferated PBMCs were stored in TC-protector medium (DS Pharma Biomedical, Osaka, Japan) at −130°C. RetroNectin/laminin/anti-CD3 antibody-coated plates were prepared before virus infection. A 1 ml mixture of RetroNectin (25 μg/ml, Takara, Shiga, Japan), laminin (10 μg/ml, Sigma-Aldrich, St. Louis, USA), and anti-CD3 antibody (5 μg/ml) in PBS was incubated in 1 well of 6-well plate at 37°C for a minimum of 3 h or at 4°C overnight. Before use, excess solution was aspirated and the 6-well plate was dried thoroughly. For modification of the differentiation medium, Activin A (100 ng/ml; R&D Systems, Minneapolis, MN, USA), Dkk (100 ng/ml; R&D Systems), Shh (200 ng/ml; R&D Systems), and Lefty-2 (500 ng/ml; R&D Systems) were added to the differentiation medium. Alternatively, RPE cells were isolated and cultured as previously described (Mayerson et al., 1985; Seko et al., 2001) and the modified differentiation medium contained 50% RPE-conditioned medium.

### Generation of Sendai virus vectors

Insert sequences containing open reading frames of human *CRX*, *NEUROD1* and *RAX* were amplified from cDNAs prepared from the total RNA of the adult human retina (Clontech Laboratories, Mountain View, CA, USA) by using Not*I*-tagged gene-specific forward and reverse primers containing SeV-specific transcriptional regulatory signal sequences that are listed in Table S1. Amplified fragments were inserted into SeV/ΔF vectors. Recovery and propagation of SeV/ΔF vectors were performed as follows. First, 293T cells were transduced with template pSeV/ΔF carrying each transgene and pCAGGS plasmids carrying the T7 RNA polymerase and NP, P, F5R, and L genes. Cells were maintained in DMEM supplemented with 10% heat-inactivated fetal bovine serum (FBS) and cultured for 1-3 days to generate the seed SeV/ΔF vector. Vectors were propagated using SeV F-expressing LLC-MK2/F7/A cells (Li et al., 2000) in MEM containing trypsin (2.5 μg/ml). Vector titers (cell infectious units/ml) of the recovered SeV/ΔF vector were determined by immunostaining using anti-SeV rabbit polyclonal serum as described previously (Fusaki et al., 2009). To generate new TS SeV vectors, mutations were introduced into conventional TS SeV/ΔF vectors (Inoue et al., 2003) by oligonucleotide-directed mutagenesis (QuikChange; Stratagene, La Jolla, CA, USA) with primer pairs listed in Table S1.

### Sendai virus infection

PBMCs were cultured at a concentration of 2×10^6^ cells/well in RetroNectin/laminin/anti-CD3 antibody-coated 6-well plates in 750 µl/well of KBM502 media for 30 min. Cells were incubated with Sendai virus solutions at approximately MOI=20 (for Fig. 1 and Fig. 4) or 50 (for Fig. 2 and Fig. 3) for 1 day, and media were then changed to differentiation media with 5% KBM502. For electrophysiological recordings, PBMCs were incubated with Sendai virus solutions at MOI=50 for 1 day. Media were changed to differentiation media without KBM502 to restrict proliferation, and recordings were performed at 5 and 6 days after transduction. For human dermal fibroblasts, after cells were cultured at a concentration of 3×10^4^ cells/well in laminin-coated 6-well plates for 1 day, Sendai virus solutions were added at adjusted MOI and cells were further incubated for 1 day, following which media were changed to differentiation media. These media were renewed every 2 days for 1 week until cells were collected for analysis.

### Retrovirus infection

Recombinant retrovirus was prepared as previously reported (Seko et al., 2012). Briefly, the full-length transcription factor *CRX* was amplified from cDNAs prepared from total RNA of adult human retina (Clontech, CA, USA) by PCR, and cloned into the *Xmn*I-*Eco*RV sites of pENTR11 (Invitrogen). The resulting pENTR11-transcription factor was recombined with pMXs-DEST by use of the LR recombination reaction as instructed by the manufacturer (Invitrogen). Retroviral DNAs were then transfected into 293T cells, and 3 days later, media were collected and concentrated. Before retroviral infection, PBMCs were cultured without FBS at least for 3 days. PBMCs were prepared at a concentration of 2×10^6^ cells/well of RetroNectin/laminin/anti-CD3 antibody-coated 6-well plates and incubated for 3 h in KBM502 media with 10% FBS. Incubated media were changed to differentiation media (DMEM/F12/B27 medium supplemented with 40 ng/ml bFGF, 20 ng/ml EGF, fibronectin, and 1% FBS) with 5% KBM502. These media were renewed every 2 days for 1 week before cells were collected for analysis. Retroviral infection of human PBMCs and dermal fibroblasts were performed as previously reported (Seko et al., 2012, 2014). To measure the efficiency of transduction by retroviral vectors, we transduced retroviral eGFP under the same conditions used for retroviral CRX. The frequency of eGFP-positive cells was approximately 80% of fibroblast cells and 40% PBMCs at 48 h after transduction by retrovirus at the same MOI.

### Reverse transcription-PCR (RT-PCR)

Total RNA was isolated with the ARCTURUS^®^ PicoPure^®^ RNA Isolation Kit (Life Technologies, Carlsbad, CA, USA) or PureLink^®^ RNA Mini Kit (Life Technologies). cDNAs were synthesized from 1 µg of total RNA with oligo (dT) primers and SuperScript^®^ III (Life Technologies). PCR was performed using GoTaq^®^ polymerase (Promega, Fitchburg, WI, USA) or KOD FX polymerase (Toyobo, Osaka, Japan). Primer sets are listed in Table S2 or in our previous report (Seko et al., 2012). Only rhodopsin was analyzed by nested PCR. These procedures were carried according to manufacturer's instructions. For all primer sets used in this study, PCR products were purified by Wizard^®^ SV Gel and PCR Clean-Up System (Promega). Direct sequencing of each PCR product was analyzed using a BigDye H Terminator Cycle Sequencing Kit (Life Technologies) and an automated capillary sequencer (3130xl Genetic Analyzer; Life Technologies). Positive or negative RT-PCR results were confirmed by duplicate experiments and representative data are shown in all figures.

### Quantitative RT-PCR

The cDNA template was amplified (ABI PRISM 7900HT Sequence Detection System, Life Technologies) using Platinum Quantitative PCR SuperMix-UDG with ROX (11743-100, Life Technologies). Fluorescence was monitored in every PCR cycle at the annealing step. The authenticity and size of PCR products were confirmed using melting curve (with software provided by Life Technologies) and gel analyses. mRNA levels were normalized using GAPDH as a housekeeping gene. The design of PCR primer sets is shown in our previous paper (Seko et al., 2012).

### Immunocytochemistry

After mixing with SeVsolutions, 50 μl PBMC suspension was dropped on a RetroNectin/laminin/anti-CD3 antibody-coated Cell Desk LF1 (Sumitomo Bakelite, Tokyo Japan) plates and incubated for 30 min. Differentiation media were added and cells were cultured for 3 days. Cells were fixed in chilled acetone at −20°C for 20 min followed by rinsing and incubated with 4% PFA in PBS for 10 min. Cells were rinsed in PBS and incubated for 1 h with mouse monoclonal anti-HN antibody (Sendai virus expressing glycoprotein, 1:5000; ID Pharma, Ibaraki, Japan) and rabbit polyclonal anti-blue opsin antibody (1:50; Abcam, Tokyo, Japan). After washing, cells were incubated with Alexa Fluor 488 conjugated anti-rabbit IgG and Alexa Fluor 568 anti-mouse IgG (1:300; Life Technologies). Nuclei were stained with DAPI and TO-PRO^®^-3 (1:1000; Life Technologies). Cells were mounted on MAS-coated glass slides (Matsunami Glass, Osaka, Japan) by using fluorescence mounting medium (Dako, Tokyo, Japan). The antibody used in Fig. 1B was rabbit anti-blue opsin (Abcam), while in Fig. S1A was goat anti-blue opsin (1:100, Santa Cruz, CA, USA). Because rabbit anti-blue opsin antibody showed stronger signal than goat, we used it for automated counting analysis. We used the goat anti-blue opsin antibody in Fig. S1A because of co-staining with anti-RAX or anti-CRX antibodies derived from rabbit. Detection of digital images and counting of immunostained cells were performed by a Nikon ECLIPSE TE300 and analyzed by NIS-Elements AR3.0 (Nikon, Tokyo, Japan).

### Light stimulation and electrophysiological recordings

For light stimulation, a high pressure UV lamp (USH-102D, Ushio, Tokyo, Japan) was used as a light source. Diffuse, unpolarized blue light was generated through bandpass filters attached to the fluorescent emission system (BX-FLA, Olympus, Tokyo, Japan). Wavelength of light used for stimulation was 460-490 nm. Duration and timing of light stimulation were controlled by an electrically controlled shutter attached to the UV lamp box. The electrically controlled shutter was triggered by commercially available software (pCLAMP 9, Axon Instruments, Foster City, CA, USA) through AD/DA. Light intensity used for stimulation was 390 W/m^2^. To activate the phototransduction cascade, 11-cis retinal (gift from the Vision Research Community, National Eye Institute, National Institutes of Health, USA) was added to culture media of human PBMCs approximately 20 min before the electrical recording (final concentration: 37.5 µM). Electrical recordings were obtained in the whole-cell patch-clamp configuration. The composition of the intrapipette solution was (in mM) KCl, 135; CaCl_2_, 0.5; HEPES, 5; EGTA, 5; ATP-2Na, 5; and GTP-3Na, 1 and pH was adjusted to 7.3 with KOH. The resistance of patch pipettes was 5-13 MΩ when filled with an intrapipette solution. The membrane current was recorded with a patch-clamp amplifier (Axopatch-200B; Axon Instruments) at 500 Hz through a DigiData 1322A interface using the pCLAMP software. Recorded data were pooled for further analysis (for details, see Fig. S4).

### Statistical analysis

Student's *t*-test or Dunnett's post hoc test (1-way ANOVA) was used for all statistical analyses (GraphPad Software Inc., La Jolla, CA, USA). For quantitative PCR, mean data (*n*=3) of non-transduced PBMCs after 6 h of cultivation were used as reference. Differences with *P*<0.05 were considered significant. In immunocytochemistry experiments (Fig. 1B), differences between means of control and blue opsin positive- or HN positive-cells were tested by Student's *t*-test (*n*=4, 1823 control cells and 2531 *CRX*-transduced cells). In time-course experiments (Fig. 1C,D), Dunnett's tests were used for multiple comparisons (*n*=3). Means+s.d. are shown in bar graphs.
